# Stokes polarimetry-based second harmonic generation microscopy for collagen and skeletal muscle fiber characterization

**DOI:** 10.1007/s10103-020-03144-6

**Published:** 2020-09-18

**Authors:** Nirmal Mazumder, Fu-Jen Kao

**Affiliations:** 1 Department of Biophysics, Manipal School of Life Sciences, Manipal Academy of Higher Education (MAHE), Manipal, Karnataka 576104 India; 2 Institute of Biophotonics, National Yang-Ming University, 11221, Taipei, Taiwan

**Keywords:** Second harmonic generation (SHG) microscopy, Polarimetry, Ultrafast optics, Collagen type I, Skeletal muscle fiber

## Abstract

The complete polarization state of second harmonic (SH) light was measured and characterized by collagen type I and skeletal muscle fiber using a Stokes vector-based SHG microscope. The polarization states of the SH signal are analyzed in a pixel-by-pixel manner and displayed through two dimensional (2D) Stokes vector images. Various polarization parameters are reconstructed using Stokes values to quantify the polarization properties of SH light. Also, the measurements are extended for different input polarization states to investigate the molecular structure of second harmonic generation (SHG) active molecules such as collagen type I and myosin.

## Introduction

The second harmonic generation (SHG) is a second-order nonlinear coherent optical process via virtual state transitions, widely used for imaging non-centrosymmetric molecules [1, 2]. The technique has been widely applied for structural imaging from the subcellular to tissue level with high penetration depth employing ultrafast pulsed lasers [3–5]. In general, due to the usage of a near-infrared ultrafast laser, it reduces scattering and absorption in bio-tissues that feature deeper imaging of penetration depth up to mm scale [6–8] and maintains high resolution and contrast as well. Most importantly, the localized excitation through a multiphoton process can greatly suppress the out-of-focus scattering, which facilitates optical sectioning capability to image a thick tissue layer by layer and then reconstructs a three-dimensional (3D) image [9]. Alternatively, SHG is a mature imaging tool for characterizing fiber orientation and morphological structure of collagen with 3D submicron spatial resolution [10, 11], in the human dermis [12, 13], keloid [1], cornea [14, 15], and the tumor microenvironment [16–19]. Furthermore, it can determine the degradation degree of type II collagen in cartilage using polarization-resolved measurements (i.e., P-SHG) [20]. SHG microscopy integrated with two-photon excitation fluorescence (TPEF) has also opened new routes towards label-free optical diagnostics [21], particularly for complex cellular assemblies of skin tissue [19] with submicron spatial resolution. It is found that SHG microscopy combined with TPEF is capable of identifying sarcomeric anomalies of skeletal muscle in physiological/biochemical state with significant sensitivity [22–25]. Additional studies found that a combination of coherent anti-Stokes Raman scattering (CARS) and SHG microscopy affords greater potential for visualizing subcellular organelles, such as mitochondria and cell nuclei, in skeletal muscle including their microstructure [26]. However, it should be noted that SH intensity generated only from non-centrosymmetric molecules and depends on the orientation of SH active molecules (collagen fiber, myosin) as well as the polarization state of excitation light [27–29].

The SHG signal from myosin and collagen is due to the protein crystal structure, mainly amino acids present in them [30]. Again, the signal strength depends on the lattice structure in three dimension (3D) and also orientation, symmetry, as well as folding of protein crystal [31]. It possesses second-order nonlinearity due to the birefringence and superhelical structures of length and width, 300 and 1 nm, respectively [32], and hence is used to discriminate the collagen in biological tissue using SHG microscopy [33, 34]. The SHG signal generation is strongly dependent on the angle between the input state of laser polarization and collagen alignment and thus the collagen orientation can be determined through the degree of polarization (DOP) [35, 36]. The quantifiable differences among sizes, shapes, and lengths of collagen fibers are important and can be automatically segmented using a suitable algorithm to speed up the analysis.

Again, a skeletal muscle fiber is formed by the spatial arrangement of myofibrils which are composed of myosin and actin filaments. These myofilaments form a cross-striated-like structure called sarcomere, which is a repeating unit of Z lines. Sarcomeres are connected serially, of length ranges from 2 to 3 μm, and the myofibrils are arranged depending on the type of muscle [5, 37]. Z line is surrounded by one section of A-band (only myosin coiled tail) and I-band (both myosin tail and head regions). It is known that myosin/actin filaments are helical and possess hexagonal and/or C6 cylindrical symmetry [20, 38, 39]. It was reported that the SHG signal is generated from the myosin head and thick filament of the sarcomere and changes the SHG intensity during contraction [28, 40, 41]. SHG image contrast is proportional to the molecular structure and order of the myosin. Also, Yuan et al. demonstrated that α- and β-myosin possess different symmetries that were observed under polarization-resolved SHG microscopy in a single-sarcomere line scan [42]. Again, the changes in the structure and physiological properties of myofibrils are investigated through the intrinsic SH signal [5, 37].

In general, Jones calculus or Stokes algebra is used for characterizing polarization light; however, only Stokes algebra is suitable for all states of polarization, partially or unpolarized [35, 43], whereas Jones calculus is only applicable for describing perfectly polarized light [44]. Conventional two-channel polarization-resolved SH microscopy is based on Jones calculus and applied to find linear birefringence and anisotropy by rotating the input polarization states and measuring the corresponding SH intensities [45–47]. We implemented Stokes vector-based polarization-resolved SHG microscopy for collagen type I and starch granule through inspection of various polarization properties [35, 36]. In this study, we describe the potential of Stokes vector-based SHG microscope to investigate the polarization properties of SH light from collagen type I and skeletal muscle fiber, where SH signal is generated from the C-N pair of the peptide bond in protein and synthetic oligopeptides [48]. We demonstrate the reconstruction of 2D Stokes vector and various polarization parameter images including the degree of polarization (DOP), linear polarization (DOLP), circular polarization (DOCP), and anisotropy.

## Materials and methods

**Fig. 1:**
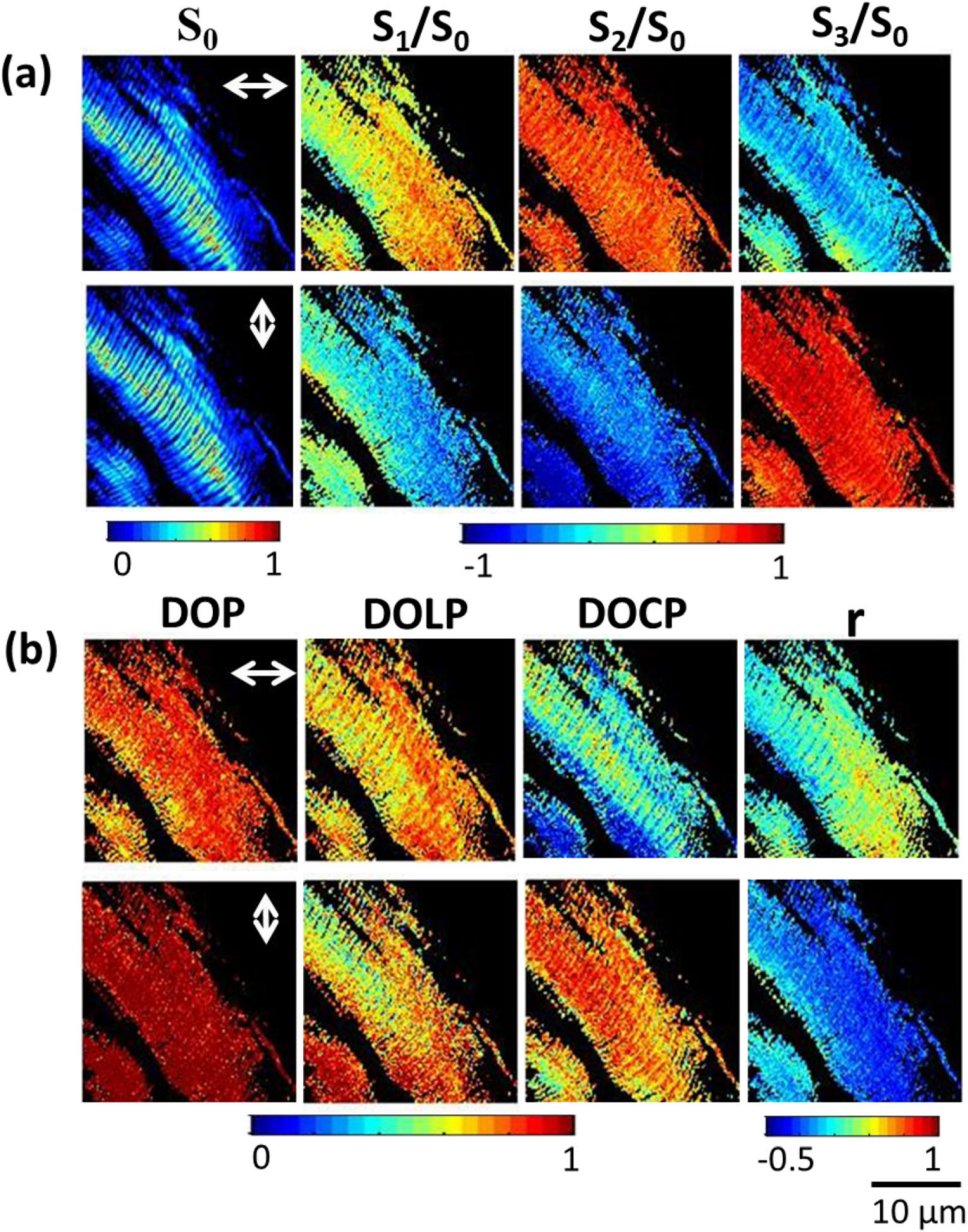
Experimental polarization-resolved SHG response from skeletal muscle fiber (a) shows the reconstructed 2D Stokes vector images and (b) represents the DOP, DOLP, DOCP, and polarization anisotropy images of SH light from the skeletal muscle fiber, when the input polarization is horizontally and vertically polarized, respectively. The color scale shows the values of each parameter

A four-channel Stokes polarimeter was built and integrated with a SHG microscope. The experimental arrangement is described in detail in [35, 36, 49]. The excitation wavelength of 800 nm from a femtosecond Ti: sapphire (Coherent Mira Optima 900-F) laser oscillator was used. The full width at half maximum (FWHM) of the laser is 15 nm of the pulse duration ~ 100 fs, with a repetition rate of ~ 76 MHz and average power of ~ 550 mW. The excitation beam is passed through the polarization state generator (PSG) which is a combination of half-wave plate and a linear polarizer. The PSG generates 0^0^ and 90^0^ polarization states of laser light. The laser beam is scanned with 50 to 50 μm scanning area to the samples mounted on an *XY* stage and focused by a × 40 objective lens (UPlanFLN N.A. 1.3, Olympus Co., Japan) [see Fig. 1, Ref. 35] of working distance in 0.51 mm. The average power of 15 mW was used at the focal plane of the objective lens. In the laboratory coordinates *XYZ*, where laser propagates in the *Z*-direction and samples are scanned in *XY*-plane, where *X* is horizontal and *Y* is vertical axis. SHG intensity is measured as a function of the relative angles between laser beam polarization and orientation of SHG-active molecule. The polarization state of laser is rotating at an angle with respect to the samples (*X*-axis). In our experiment, collagen fiber and skeletal muscle are thick and arbitrary oriented and hence we assumed them to be parallel to *XY*-plane. The forward directed SH signal is collected using a × 20 objective lens (0.75 N.A., Olympus Co., Japan). A 400 ± 40 nm (Edmund Optics Inc., USA) bandpass filter along with a 680-nm short pass (Semrock) filter was also inserted into the SHG emission path for SH signal collection. The SH light is passed through a polarization state analyzer (PSA) to reconstruct the Stokes parameters [36]. The SH signals are guided through liquid light guides (5-mm core diameter, LLG0538-6, Thorlabs Inc.) to four photo-multiplier tubes (PMTs). Four PMTs were integrated with time-correlated single-photon counting electronics (TCSPC, PicoHarp300, PicoQuant GmbH, Germany) for measuring the SH signals simultaneously. The SHG Stokes vector “S_out_” is measured from the four intensity images, *I*, using the equation: *S*_out_ = (*A*_4 × 4_)^−1^ ⋅ *I*. MATLAB (MathWorks, R2009b, Natick, MA) program is used to reconstruct the 2D Stokes vector and various polarization parameters [35]. The SH signals are measured from collagen type I and skeletal muscle fiber (attached at both ends to a coverslip) for 0^0^ and 90^0^ polarization states of laser light. The tail of the adult mouse was removed and immediately incubated in liquid nitrogen. The tail was dissected and individual fibers were extracted by microdissection. The imaging experiment was performed immediately after fiber extraction.

## Results and discussion

**Fig. 2:**
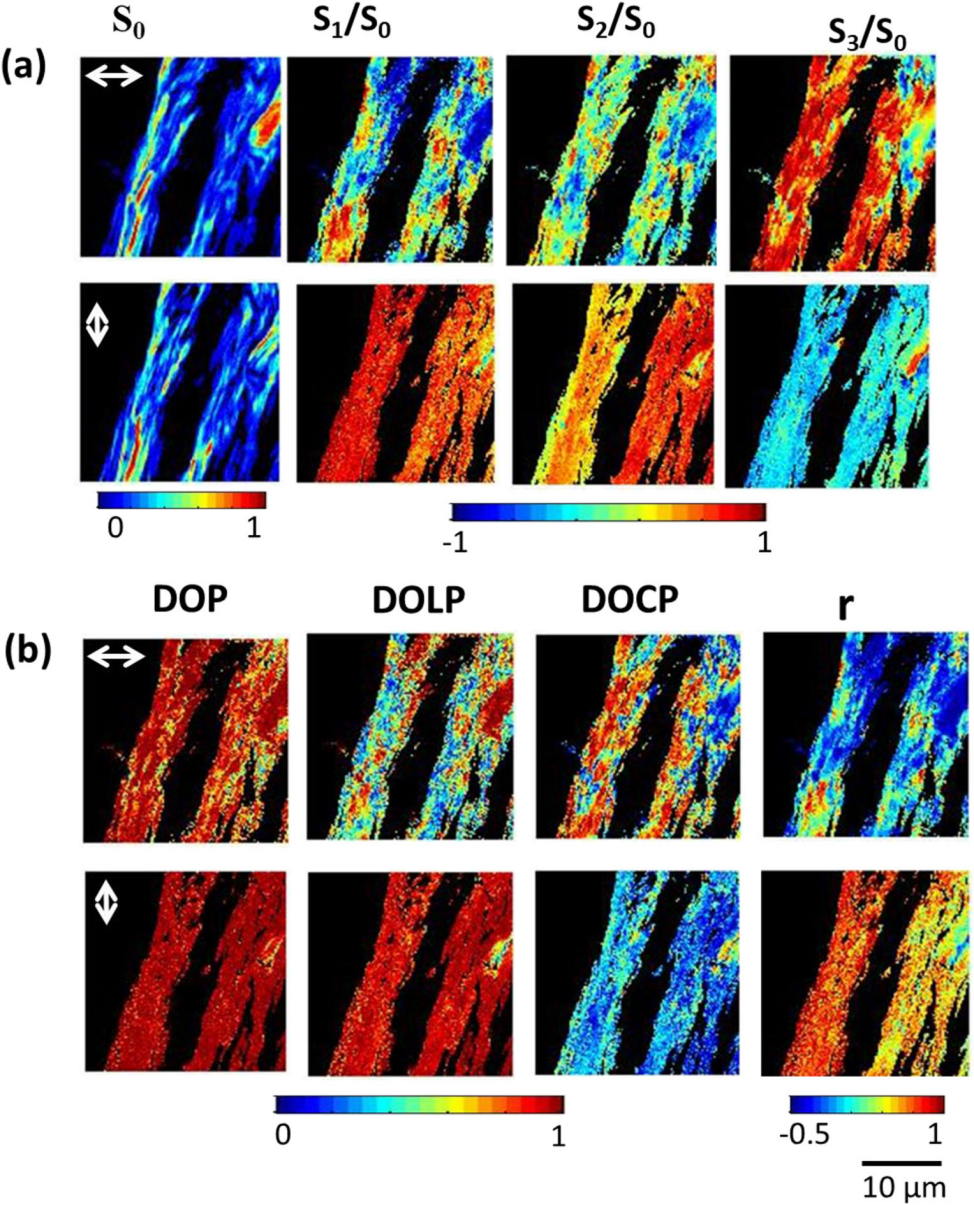
Experimental polarization-resolved SHG response from collagen fiber (a) shows the reconstructed 2D Stokes vector images and (b) represents the DOP, DOLP, DOCP, and polarization anisotropy images of SH light from the collagen fiber, when the input polarization is horizontally and vertically polarized, respectively. The color scale shows the values of each parameter

Stokes parameters of SH signal from collagen type I and skeletal muscle fiber are measured with various input laser polarization states to investigate the alignment and orientation of fibers. Polarization microscopy improves the image contrast of sarcomeres in myofibrils due to the linear birefringence; the origin of SHG contrast arises from a quadratic dependence on the SHG active protein concentration. Previously, it was reported that SH contrast is enhanced with selective arrangements of collagen type I and muscle fibers [36, 50]. Figure 1(a) and Fig. 2(a) shows the 2D Stokes vector SHG images from skeletal muscle fiber and collagen type I with different input laser beam polarizations. Figure 1(b) and Fig. 2(b) show the spatial variation of the DOP, DOLP, DOCP, and anisotropy, *r*, as was derived from the four Stokes parameters from the samples, shown in Figs. 1(a) and Fig. 2(a).

Figure 1 shows the SHG images of the myosin in muscle fiber. The polarization-dependent SHG images depict the high second-order nonlinearity due to densely packed fibrous protein myosins. We have reconstructed 2D Stokes vector SHG images of skeletal muscle fibers with various input polarization states of light (as shown in Fig. 1). The orientation distribution can be determined by measuring the different Stokes parameters along with the intensity contrast in the image. We can also identify the isotropic (I) and anisotropic (A) bands in sarcomeres, which are displayed as an alternating dark and bright bands in the SHG image which helps in measuring the length of the sarcomere of ~ 3 μm. The myofibrils are well packed and interconnected serially through intermediate filaments called sarcomeres. The dependency of the SH signal on the direction of the polarization state of the incident beam of light was hence examined. In Fig. 1(b), the DOP value is higher for vertically polarized light illumination. The higher values of DOP show that myofibrils are packed parallel to the given direction and those sarcomeres are in phase with each other. On the other hand, the DOP value tends to be lower when the polarization of the laser is horizontally polarized. In Fig. 1(b), changes in the DOLP values are attributed to the signal depolarization, whereas the changes in the DOCP of SH light are due to a combination of depolarization and muscle birefringence. DOLP values are equal to 1 corresponding to perfectly linear polarized light, whereas DOLP equal to 0 corresponds to depolarized light. The DOLP image clearly shows that the DOLP values are higher for 0^0^ polarized excitation than the 90^0^ polarized excitation, which is due to the strong SH signal dependency on the direction of the input polarization. DOCP values also varied significantly for two different excitation polarization since the structural arrangement of sarcomere bands are different. Furthermore, the molecular arrangement of microfibrils in skeletal muscle is measured by anisotropy “r” and ranges from − 0.5 to 1. The anisotropy images show that molecules are better aligned 90^0^ since the “r” value is approximately − 0.5 when 90^0^ polarized incident light. However, in the case of 0^0^ polarized excitation light, the anisotropy value is approximately 0. Thus, it is clearly shown that the SH signal varies significantly with different excitation polarization angles. Figure 2(a) and 2(b) shows the normalized Stokes parameters and reconstructed various polarization parameters of SHG images of collagen type I fiber when the incident beam is 0^0^ and 90^0^ polarized, respectively. The SHG images are acquired at a depth of 20 μm. Stokes parameters are varied with different input polarization states which are due to the arrangement of the optical axis in collagen fibers. The polarization parameters are analyzed in each pixel of the image; the DOP value is approximately 1 indicating the SH signal is fully polarized. The DOLP values show the alignment of fiber and molecules parallel to the vertically/horizontal polarization state of the input laser beam. DOCP determines how the coil-like structure of collagen type I changes the relative polarization between the incident fundamental and SH signal. The anisotropy measurements of the SHG signal exploit the effect of birefringence, which is due to the sample heterogeneity in the Z propagation direction from collagen type I.

## Conclusion

We discuss a Stokes polarimeter in combination with SHG microscopy to fully characterize the polarization dependence of SH signal. The polarization states of SH signal are measured, analyzed in each pixel of the 2D images, and displayed through Stokes vectors. Various polarization parameters are reconstructed using Stokes values to quantify the polarization properties of SH light. The Stokes vector-based analysis demonstrated that molecular arrangements in collagen type I and skeletal muscle fiber are different with high spatial resolution. The effect of birefringence and crystal orientation of skeletal muscle fiber and collagen type I are visualized from the polarization parameters. We observed that DOP is approximately unity at 90^0^ polarizations in both the samples, which indicates that SH active molecules are parallel to the 90^0^ laser polarization. The technique has the potential in the field of biomedical applications where the structure or orientation of molecules is a key factor including the progress of wound healing and tissue morphology.

The developed four-channel-based Stokes vector-based SHG microscopy measures the full Stokes parameters of the SHG light in transmission geometry from thin samples such as starch, collagen, and skeletal muscle fiber. In case of thick tissue samples, the penetration depth of laser is limited and SHG light intensity is weak and therefore cannot be collected in the forward direction. In the case of in vivo imaging, SHG light is generated from the surface of the image plane and detected in epi-detection. The Stokes vector-based measurement can be implemented in an epi-detection mode for in vivo imaging with a slight modification of optical design. The Stokes polarimeter should be placed in epi-detection for SHG signal collection although fiber optics-based endoscopy integrated with SHG microscopy is demonstrated for clinical applications [51, 52].
